# A Serious Impediment in the Speech Removed by a Simple Operation upon the Teeth

**Published:** 1841

**Authors:** E. Maynard


*A serious impediment in the speech removed by a simple operation upon the teeth.*—By E. Maynard, M. D.

In May, 1840, I separated the superior central incisors of Maj. W. of this city, for the removal of incipient caries. A few days afterwards he informed me that the operation had been doubly beneficial, as *it had perfectly removed a very troublesome impediment in his speech which had afflicted him from infancy!* When articulating a word beginning with '*th*' his tongue would adhere to the superior incisors, and the roof of his mouth so firmly as to render it necessary to drop his chin before he could pronounce the required sound. Separating the central incisors opened an *air passage* to the roof of the mouth, above the end of the tongue. This prevented the formation of a vacuum there, and of course relieved the tongue from atmospheric pressure—the apparent cause of the difficulty.

*Washington, Feb.* 20, 1841.

